# Etiology and Outcomes of Healthcare-Associated Meningitis and Ventriculitis—A Single Center Cohort Study

**DOI:** 10.3390/idr14030045

**Published:** 2022-06-03

**Authors:** Hana Panic, Branimir Gjurasin, Marija Santini, Marko Kutlesa, Neven Papic

**Affiliations:** 1 School of Medicine, University of Zagreb, Šalata 3b, 10000 Zagreb, Croatia; hanaapanic@gmail.com (H.P.); msantini@bfm.hr (M.S.); mkutlesa@bfm.hr (M.K.); npapic@bfm.hr (N.P.); 2 Department for Adult Intensive Care and Neuroinfections, University Hospital for Infectious Diseases Zagreb, Mirogojska 8, 10000 Zagreb, Croatia; 3 Department for Viral Hepatitis, University Hospital for Infectious Diseases Zagreb, Mirogojska 8, 10000 Zagreb, Croatia

**Keywords:** healthcare-associated meningitis and ventriculitis, postoperative meningitis, HCAMV, nosocomial infections, *Acinetobacter baumannii*

## Abstract

Healthcare associated meningitis and ventriculitis (HCAMV) are serious complications of neurosurgical procedures. We conducted a retrospective cohort study of patients with HCAMV treated at the University Hospital for Infectious Diseases Zagreb during the 2013–2019 period. A total of 144 patients with 151 episodes of HCAMV were included. The most common indications for neurosurgical procedures were brain tumor, hemorrhage and hydrocephalus. Etiology was identified in 90 (59.6%) episodes (either positive CSF culture or positive PCR), and in other 61 (40.39%) the diagnosis of HCAMV was made based on clinical and CSF parameters, without microbiologic confirmation. Carbapenem-resistant Acinetobacter baumannii was the most common pathogen (15.89%), followed by Staphylococcus aureus (13.91%), Pseudomonas aeruginosa (13.25%) and Coagulase negative staphylococci (7.95%). Overall, 24 (16.3%) patients died, and the majority had adverse outcomes, persistent vegetative state (8, 5.56%) and severe disability (31, 21.53%). The worst clinical outcomes were observed in A. baumannii infections. High rate of complications, the need for external ventricular drainage (re)placement often complicated with nosocomial infections and prolonged stay in intensive care units were observed. Clinicians should be aware of local microbial epidemiology on guiding proper empirical antimicrobial treatment in patients with HCAMV.

## 1. Introduction

Healthcare associated meningitis and ventriculitis (HCAMV) are serious complications in patients undergoing neurosurgical procedures, resulting in longer hospital stay and high rates of adverse outcomes [1]. Reported incidence of HCAMV varies widely, usually ranging between <1% and 10% of patients undergoing neurosurgical procedures [1,2,3]. Risk factors include invasive procedures (such as craniotomy, ventricular catheters), head trauma, hemorrhage and less commonly metastatic infection [1,2,3]. Nonspecific symptoms and cerebrospinal fluid (CSF) findings might be difficult to distinguish from the underlying neurological disease or postsurgical-related conditions. Consequently, diagnostic delay contributes to severe morbidity and mortality [1]. While Gram-positive bacteria have traditionally been considered as the leading cause of HCAMV, recently the number of Gram-negative cases has largely increased with the predominance of multidrug-resistant (MDR) pathogens [4,5,6]. The studies on HCAMV show significant variability and are difficult to compare given the differences in local microbiology, case definitions, infection prevention and treatment protocols. The aim of this study was to describe clinical and microbiological findings, complications and outcomes of HCAMV.

## 2. Materials and Methods

A retrospective, cohort study included all consecutively hospitalized adult patients with HCAMV in the University Hospital for Infectious Diseases Zagreb, Croatia (UHID) between 2013 and 2019. The following parameters were collected from patients’ charts: demographic data, clinical presentation, comorbidities, baseline clinical and neurological status, CSF profile (White blood cell count (WBC), glucose, and protein), routine serum and microbiological tests. Clinical progression, complications, mortality and functional status measured by Glasgow outcome score (GOS) at hospital discharge were analyzed. Patients were diagnosed with HCAMV according to current guidelines [7]. Briefly, the patients were required to have one of the following criteria: (1) organism cultured from CSF; and (2) at least two of the following symptoms with no other recognized cause: fever > 38 °C or headache, meningeal signs, or cranial nerve signs and at least one of the following: (a) increased WBC, elevated protein, and decreased glucose in CSF; (b) positive CSF Gram stain; (c) positive CSF PCR test; or (d) positive blood culture. Data are presented descriptively with frequencies and medians with first and third quartile (IQR). Binary logistic regression analysis was used to assess the independent predictors of adverse outcomes, defined as GOS 1 to 3. Statistical analyses were performed using the GraphPad Prism Software version 9.1.1. (San Diego, CA, USA).

## 3. Results

A total of 144 patients (with 151 HCAMV episodes) were included; 91 (63.19%) males, median age of 53 (IQR 35–66) years. Seven patients had two episodes of HCAMV; all of them had ventricular drain infection, and they were considered as separate episodes since clinical improvement and normalization of CSF preceded a new onset of clinical and CSF deterioration with at least two weeks apart. The most common indications for neurosurgical procedures are presented in Table 1. Forty (27.78%) patients had more than one indication for neurosurgical treatment (most commonly trauma with hemorrhage in 19 and brain tumor with hydrocephalus in 11 patients). External ventricular (EVD) or ventriculoperitoneal (VP) drain was placed in 24 (16.67%) patients.

The median time from neurosurgical procedure to HCAMV diagnosis was 7 (IQR 3–14) days. The majority of patients presented with fever, headache, changes in mental status or focal neurological deficit, while meningeal symptoms and new onset seizures were less commonly observed (Table 1). Serum C-reactive protein was elevated in 116 patients with a median of 57 mg/L (IQR 20–113), and leukocytosis was present in 75 patients (median of 11.5 × 10^9^/L, IQR 8.4–14.5). All patients had abnormal CSF findings; CSF WBC count of 431/mm^3^ (80–1850), protein 1.6 g/L (1.00–2.9) and glucose 2.1 mmol/L (1.1–3.1).

Etiology was identified in 90 (59.6%) episodes: CSF culture was positive in 56 of 118 (47.46%), Gram stain in 19 of 91 (20.87%), 16S rDNA PCR in 10 of 75 (13.33%); 11 of 128 (8.59%) had concurrent bacteremia. *Acinetobacter baumannii* was the most common pathogen (all isolates were resistant to carbapenems), followed by *P. aeruginosa*, MSSA, CoNS and MRSA, as presented in Table 2. Twenty-eight patients (18.54%) had polymicrobial infections. The most common co-infections were due to Gram negative bacteria + CoNS (*n* = 5), *S. aureus* + CoNS (*n* = 2), or *A. baumannii* + other Gram-negatives (*n* = 4). *Candida albicans* was isolated in three patients, two of them were later diagnosed with neurotuberculosis.

Regarding laboratory findings in the context of etiological diagnosis, patients with CoNS isolated in monoculture had the lowest CSF WBC count (86/mm^3^, IQR 21–427) and proteinorachia (0.97 g/L, IQR 0.28–1.72), while patients with *P. aeruginosa* and *A. baumannii* had higher pleocytosis (1280/mm^3^, IQR 276–1973 and 512, IQR 240–3347, respectively) and proteinorachia (2.0 g/L, IQR 1.1–4.0 and 1.7, IQR 1.4–4.9, respectively).

The majority of patients (118, 81.94%) were admitted to the intensive care unit (ICU); 37 (25.69%) required mechanical ventilation for the median duration of 12 (IQR 6–28) days. Nine patients required renal replacement therapy. During HCAMV treatment EVD was placed in 65 (45.14%) and was replaced due to complications in 29 patients (median of 2 EVD placement per patient). Twenty-seven patients (18.75%) had a complication of a newly acquired meningitis associated with EVD. EVD was subsequently converted to VP in 25 (17.48%) patients.

**Figure 1:**
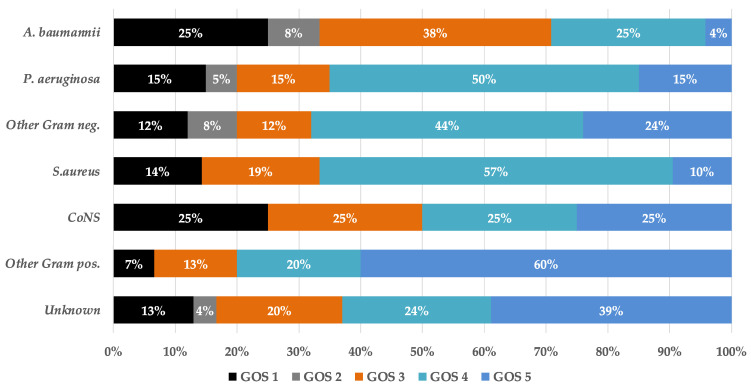
The stratified clinical outcomes by etiology expressed as Glasgow outcome score (GOS).

The median duration of hospitalization was 27 (IQR 16–44) days and ICU stay of 20 (IQR 13–31) days. Twenty-four (16.3%) patients died, and the majority had adverse outcomes: persistent vegetative state in 8 (5.56%) and severe disability in 31 (21.53%). At hospital discharge 39 (27.08%) had moderate disability and 42 (29.17%) had good recovery. The stratified clinical outcomes by etiology are displayed in Figure 1. The worst clinical outcomes were observed in *A. baumannii* infections, followed by CoNS. On logistic regression analysis, Glasgow coma score < 8 (OR 6.2, 95%CI 1.67–23.02), mechanical ventilation (OR 16.88, 95%CI 5.06–56.27), newly acquired meningitis associated with EVD (2.99, 95%CI 1.10–8.14) and hemorrhaging event that led to surgery (2.29, 95%CI 1.01–5.26) were associated with adverse clinical outcomes, defined as GOS of 1–3.

## 4. Discussion

Diagnosis of HCAMV is often delayed due to non-specific symptoms (meningeal symptoms are relatively rare), pre-existing inflammation (alternating the CSF profile) and antecedent antimicrobial use.

In our study, the majority of patients had some clinical signs or abnormal CSF findings that could be related to HCAMV, leading to a high rate of suspicion. However, the CSF cytologic and biochemical profile can be drastically altered by trauma, hemorrhage, postoperative aseptic inflammation, or EVD [8,9,10]. While pleocytosis implies the presence of infection, normal CSF WBC does not exclude it. In a large retrospective cohort, 20% of patients with shunt-meningitis had normal CSF WBC, CSF proteins in 42% and CSF-to-blood glucose ratio in 48% [11].

Therefore, microbiologic confirmation is essential for diagnosis and management. However, the Gram stain and CSF culture have limited sensitivity and reported negativity rates varied from 10% to 40% (in our study 40% of episodes were etiologically negative) [2,4,6,12,13]. This might be associated with antibiotic use prior to CSF sampling, inclusion of only culture positive patients, or over-valorizing clinical and CSF features due to meningeal reactions to trauma or cerebral hemorrhage.

Our study demonstrated the potential of 16S rRNA gene PCR/sequencing (30% of tested, culture negative CSF samples were positive) that can identify the presence of bacteria even in patients who have already received antibiotics [14,15,16]. This method can also provide more rapid identification, especially of slow-growing and fastidious bacteria [17,18]. Multiple studies on broad range PCR methods in HCAMV report high specificity and often limited sensitivity [19,20,21]. Multiplex PCR methods may also increase diagnostic yield, but are limited with predefined bacterial panels which might not include pathogens prevalent in some settings [22,23]. Current guidelines state that more studies are needed before routine use of PCR can be recommended [7]. Here, we described the largest cohort of patients in which this method has been clinically utilized in conjunction with culture and Gram stain examination.

MDR *A. baumannii*, *P. aeruginosa* and staphylococci were the most common causes of HCAMV in our cohort. The changing etiology with predominance of Gram-negative bacteria has already been reported in some countries [4,13,24,25]. Better adherence to antibiotic prophylaxis directed against Gram-positive bacteria and rising prevalence of MDR Gram-negative bacteria in hospitals contribute to this shift.

In our study, overall mortality rate was 16.3%, which is in the range of previous reports [2,12,24,26]. However, Gram-negative HCAMV was associated with worse clinical outcomes. Several studies from centers with high prevalence of MDR HCAMV showed significantly higher mortality in these patients [2,24,27]. Senturk G.C. et al. in their retrospective case-control study (*n* = 112) reported significantly higher mortality among patients with *A. baumannii* HCAMV (56% vs. 24%) [24]. Similarly, in a Brazilian cohort the reported mortality was 73% [28]. In a systematic review that included 899 HCAMV episodes, overall mortality was 27%; 55% in *A. baumanii* group compared to 19% in patients with *S. aureus* [5].

CoNS had the second worst outcome (after *A. baumannii*), which may be linked with intracranial devices/drains, late diagnosis (less prominent CSF changes) and more indolent clinical course [27]. Some reported better outcomes in CoNS HCAMV when compared to Gram-negative HCAMV [29]. This might be due to absence of *A. baumannii* infections and the low prevalence of other MDR Gram-negative pathogens in the reporting clinic. Gram-negative HCAMV had more prominent CSF changes, in contrast to some studies which showed no differences in CSF leukocyte counts [2,24,27].

The rise of MDR *A. baumannii* HCAMV cases is concerning due to limited antimicrobial options and almost universal resistance to carbapenems (all in our cohort). Hence, polymyxins are often the only therapeutic option, however with low CSF penetration not adequate for bactericidal activity and potentially serious adverse effects [30].

High rate of complications, need for EVD (re)placement often complicated with new infections and prolonged ICU stay were observed, highlighting the burden HCAMV can have on healthcare systems. Notably, adverse outcomes were frequent; at hospital discharge, 54.17% of patients were either in persistent vegetative state or had significant disability. This was also pathogen-specific in our study. A cohort from Texas with low MDR prevalence showed similar outcomes (50% of patients had GOS 2 or 3) [31]. In Portuguese cohort 24% of patients at discharge had GCS < 13 with in-hospital mortality rate of 37% [25]. Importantly, very few of the studies report outcomes other than mortality or duration of hospitalization, therefore the true impact of HCAMV on outcomes after hospital discharge might be underestimated.

While current guidelines recommend combined therapy with vancomycin and an anti-pseudomonal β-lactam for empirical treatment [7], taking local microbiological data in account is of the highest importance, and in some regions the addition of a polymyxin may be necessary. Given the relatively scarce published data on MDR HCAMV, studies as our have practical implications since they give insight into optimal empirical treatment.

The main limitation of this study arises from its retrospective design; the impact of antimicrobial prophylaxis and choice or duration of treatment were not evaluated due to high heterogeneity of our cohort; long-term follow-up was not undertaken. Indeed, quality studies comparing antimicrobial approaches in different neurosurgical scenarios are needed.

## 5. Conclusions

In conclusion, HCAMV was associated with MDR pathogens, specifically Gram-negative rods, prolonged hospitalization and ICU stay, occurrence of complications and poor clinical outcomes. This highlights the importance of awareness of the local microbiological epidemiology, adequate empirical therapy and implementation of infection prevention and control programs.

## Figures and Tables

**Table 1:** Baseline patients’ characteristics.

Characteristics	Number of Patients (%) or Median (IQR)
Median age, years	53 (35–66)
Male sex	91 (63.19%)
Female sex	53 (36.81%)
Immunocompromised	16 (11.11%)
**Indication for neurosurgical procedure**	
Hemorrhage	54 (37.50%)
Subarachnoid	29 (20.14%)
Intraventricular	10 (6.94%)
Intracerebral	15 (10.42%)
Hydrocephalus	42 (29.17%)
Trauma	26 (18.06%)
Brain tumor	72 (50.00%)
Other	15 (10.42%)
Presence of ventriculoperitoneal (VP) shunt or external ventricular (EVD) before infection	24 (16.67%)
**Clinical presentation**	
Time from neurosurgery, days	7 (3–14)
Fever	100 (69.44%)
Glasgow coma score ≤ 14	63 (43.73%)
Glasgow coma score ≤ 8	28 (19.44%)
Headache	73 (50.69%)
Changes in mental status	83 (57.64%)
Nausea/vomiting	48 (33.33%)
Focal neurological deficit	74 (51.39%)
Neck stiffness	51 (35.42%)
Seizures	20 (13.89%)
Photophobia	15 (10.42%)
Cerebrospinal fluid leak	27 (18.75%)

**Table 2:** Etiology of healthcare associated meningitis and ventriculitis.

Etiology	Number of Episodes (%)
Etiology unknown	61 (40.39%)
**Gram-positive bacteria**	
*Staphylococcus aureus*	21 (13.91%)
Methicillin-resistant *Staphylococcus aureus* (MRSA)	16 (10.60%)
Methicillin-susceptible *Staphylococcus aureus* (MSSA)	5 (3.31%)
Coagulase-negative *Staphylococcus* (CoNS)	12 (7.95%)
*Streptococcus* spp.	6 (3.97%)
*Enterococcus* spp.	5 (3.31%)
*Rothia mucilaginosa*	1 (0.66%)
*Corynebacterium* spp.	2 (1.32%)
*Bacillus* spp.	1 (0.66%)
*Cutibacterium acnes*	2 (1.32%)
**Gram-negative bacteria**	
*Pseudomonas* spp.	20 (13.25%)
*Enterobacter* spp.	8 (5.30%)
*Klebsiella pneumoniae*	7 (4.64%)
*Acinetobacter baumannii*	24 (15.89%)
*Escherichia coli*	4 (2.65%)
*Citrobacter* spp.	3 (1.99%)
*Serratia marcescens*	2 (1.32%)
*Fusobacterium nucleatum*	1 (0.66%)
**Other microorganisms**	
*Candida albicans*	3 (3.99%)
*Mycobacterium tuberculosis*	2 (1.32%)
Mixed infection	28 (18.54%)

## Data Availability

The datasets generated during and/or analyzed during the current study are available from the corresponding author on reasonable request.
